# Implementation of a pragmatic, stepped-wedge cluster randomized trial to evaluate impact of Botswana’s Xpert MTB/RIF diagnostic algorithm on TB diagnostic sensitivity and early antiretroviral therapy mortality

**DOI:** 10.1186/s12879-016-1905-4

**Published:** 2016-10-26

**Authors:** Andrew F. Auld, Tefera Agizew, Sherri Pals, Alyssa Finlay, Ndwapi Ndwapi, Rosanna Boyd, Heather Alexander, Anikie Mathoma, Joyce Basotli, Sambayawo Gwebe-Nyirenda, James Shepherd, Tedd V. Ellerbrock, Anand Date

**Affiliations:** 1 Division of Global HIV & TB, Center for Global Health, United States Centers for Disease Control and Prevention (CDC), 1600 Clifton Road, Atlanta, GA 30333 USA; 2 Centers for Disease Control and Prevention Botswana, Plot 14818 Lebatlane Road, Gaborone, Botswana; 3 Division of TB Elimination, National Center for HIV, Hepatitis and STD Prevention, Centers for Disease Control and Prevention, 1600 Clifton Road, Atlanta, GA 30333 USA; 4 Ministerial Strategy Office, Ministry of Health, 24 Amos Street, Gaborone, Botswana; 5 Yale University School of Medicine, 333 Cedar St, New Haven, CT 06510 USA

**Keywords:** Xpert MTB/RIF, Diagnostic accuracy, Sensitivity, Antiretroviral therapy, People living with HIV, Mortality, Stepped-wedge cluster randomized trial, Botswana

## Abstract

**Background:**

In 2012, as a pilot for Botswana’s national Xpert MTB/RIF (Xpert) rollout plans, intensified tuberculosis (TB) case finding (ICF) activities were strengthened at 22 HIV treatment clinics prior to phased activation of 13 Xpert instruments. Together, the strengthened ICF intervention and Xpert activation are referred to as the “Xpert package”.

**Methods:**

The evaluation, called the Xpert Package Rollout Evaluation using a Stepped-wedge design (XPRES), has two key objectives: (1) to compare sensitivity of microscopy-based and Xpert-based pulmonary TB diagnostic algorithms in diagnosing sputum culture-positive TB; and (2) to evaluate impact of the “Xpert package” on all-cause, 6-month, adult antiretroviral therapy (ART) mortality. A pragmatic, stepped-wedge cluster-randomized trial design was chosen. The design involves enrollment of three cohorts: (1) cohort R, a retrospective cohort of all study clinic ART enrollees in the 24 months before study initiation (July 31, 2012); (2) cohort A, a prospective cohort of all consenting patients presenting to study clinics after study initiation, who received the ICF intervention and the microscopy-based TB diagnostic algorithm; and (3) cohort B, a prospective cohort of all consenting patients presenting to study clinics after Xpert activation, who received the ICF intervention and the Xpert-based TB diagnostic algorithm. TB diagnostic sensitivity will be compared between TB culture-positive enrollees in cohorts A and B. All-cause, 6-month ART-mortality will be compared between cohorts R and B. With anticipated cohort R, A, and B sample sizes of about 10,131, 1,878, and 4,258, respectively, the study is estimated to have >80 % power to detect differences in pre-versus post-Xpert TB diagnostic sensitivity if pre-Xpert sensitivity is ≤52.5 % and post-Xpert sensitivity ≥82.5 %, and >80 % power to detect a 40 % reduction in all-cause, 6-month, ART mortality between cohorts R and B if cohort R mortality is ≥13/100 person-years.

**Discussion:**

Only one small previous trial (*N* = 424) among ART enrolees in Zimbabwe evaluated, in a secondary analysis, Xpert impact on all-cause 6-month ART mortality. No mortality impact was observed. This Botswana trial, with its larger sample size and powered specifically to detect differences in all-cause 6-month ART mortality, remains well-positioned to contribute understanding of Xpert impact.

**Trial registration:**

Retrospectively registered at ClinicalTrials.gov: NCT02538952.

**Electronic supplementary material:**

The online version of this article (doi:10.1186/s12879-016-1905-4) contains supplementary material, which is available to authorized users.

## Background

In Botswana, as in the rest of sub-Saharan Africa, undiagnosed tuberculosis (TB) or TB diagnosed late in the course of disease is thought to be the most common cause of death among persons living with HIV (PLHIV), whether they are receiving antiretroviral therapy (ART) or not, with TB accounting for about 40 % of deaths according to a recent meta-analysis of pathological autopsy studies [1]. Although antiretroviral therapy (ART) reduces risk of all-cause mortality among PLHIV, early mortality in the first 3–6 months after ART initiation remains high in sub-Saharan Africa and is commonly due to undiagnosed TB or TB diagnosed late [2–4].

Reasons for failure to diagnose TB early among PLHIV can be categorized as patient-related or health facility-related. Patient-related reasons include: (1) failure to present to a health facility when symptoms arise, which may be due to poor access to health care or cultural norms that delay health-seeking behavior [5], and (2) absence of TB symptoms among late presenters with advanced immune suppression because TB symptoms are dependent on both bacillary burden and immune response [6].

Healthcare facility-related reasons for missed or late TB diagnoses among PLHIV include: (1) failure of healthcare workers (HCW) to screen for TB symptoms [7–9]), (2) failure of HCWs to request sputum or other diagnostic samples from symptomatic patients [10], (3) inability to collect high quality sputa or other appropriate diagnostic samples from symptomatic patients [11], (4) insensitive TB diagnostics, with smear microscopy alone having a sensitivity of about 45 % in diagnosing culture-positive disease among PLHIV [12], (5) inability to diagnose drug-resistant TB timeously [13], and (6) long turn-around times for some TB diagnostic tests or failure to return results to clinicians and patients [14].

In 2009, the commercial release of the Xpert MTB/RIF assay (Xpert) for the GeneXpert platform represented an important breakthrough in TB diagnostics. With features including sensitivity of about 79 % in diagnosing culture-positive TB from sputum samples among PLHIV [15], significantly superior to smear microscopy [12], ability to detect rifampicin resistance-conferring mutations, capacity to run the test on sputum samples within 100 min after brief sample processing, and minimal laboratory training requirements, Xpert significantly advanced TB diagnostic capability for clinicians managing PLHIV, especially in resource-limited settings (RLS) [16]. However, Xpert on its own, cannot solve all the facility-level challenges to diagnosing TB [17]. Strengthening of the entire TB symptom screening and diagnostic algorithm is needed for Xpert to have maximum impact on patient health outcomes in most RLS [17].

Therefore, in 2012, the Botswana Ministry of Health (MOH) and the United States Centers for Disease Control and Prevention (CDC), designed a package of intensified TB case finding (ICF) interventions to be rolled out prior to, and in coordination with, the phased activation of 13 Xpert devices in support of 22 HIV care and treatment clinics.

The package of ICF interventions included: (1) ensuring the 22 HIV care and treatment clinics adopted the World Health Organization (WHO)-recommended four-symptom TB screen for adults (>12 years old as defined by the HIV care and treatment program); (2) situating trained TB case-finding nurses in all 22 facilities to implement the screening and diagnostic algorithms; and (3) training TB case-finding nurses and other health facility personnel in both smear-microscopy-based and Xpert-based TB diagnostic algorithms for adults and children. The combination of the ICF interventions and rollout of the Xpert device is referred to as the “Xpert package” in this report.

To evaluate the accuracy of the new MOH-proposed Xpert diagnostic algorithm and also the impact of the whole Xpert package on patient outcomes, a pragmatic, stepped-wedge cluster-randomized trial (CRT), referred to as the Xpert Package Rollout Evaluation using a Stepped-wedge design (XPRES), was initiated. In this paper, the protocol-specified key study objectives, design rationale, sample size, key procedures, and analytic approaches are described. In addition, the evolution of power estimates over time as real-time study enrollment numbers became available, and key amendments to study procedures, which were needed to adapt to operational challenges, are described.

## Methods

### Key objectives

The first objective of the evaluation is to determine whether the new MOH-recommended Xpert-based pulmonary TB diagnostic algorithm (including Xpert testing of sputum samples for all patients screening positive (i.e., presumptive TB patients) and chest x-ray for Xpert-negative presumptive TB patients) is more sensitive than the pre-Xpert smear-microscopy-based algorithm (smear microscopy and chest x-ray for smear-negative presumptive TB patients) in diagnosing culture-positive TB disease among adult PLHIV. Although it is expected that the Xpert-based TB diagnostic algorithm will be both more sensitive and more specific than the pre-Xpert algorithm, superiority of the Xpert *algorithm* has not yet been demonstrated in Botswana and thorough evaluation of the accuracy of the new diagnostic algorithm is important to guide future investments [18, 19].

The second objective is to evaluate the impact of the whole Xpert package on all-cause mortality during the first six months of ART, among adult PLHIV. With an estimated 40 % of early ART deaths due to undiagnosed TB or TB diagnosed late, the Xpert package could conceivably reduce all-cause, 6-month ART mortality by ensuring: (1) that all ART enrollees are appropriately screened for TB symptoms before and during ART, and (2) that presumptive TB patients have access to a sensitive TB test (Xpert) and early TB treatment where warranted [20]. Only one small trial (*N* = 424) in Zimbabwe has previously aimed to examine the impact of Xpert-versus microscopy-based TB diagnostic algorithms on 6-month ART mortality [21]; no difference in early ART mortality was noted between study arms, however, the small sample size and high rates of empiric TB treatment in both study arms limit study findings.

### Study design rationale

A pragmatic stepped-wedge CRT design was chosen because: (1) Xpert device activation was most feasibly achieved for an entire district TB laboratory, which often served more than one health facility; this fact made an individual randomized controlled trial (RCT) design less desirable [19], (2) according to WHO guidance [22] and MOH guidelines [23], the Xpert device was expected to be beneficial for both patients and providers, and therefore it was considered ethically sub-optimal to implement a parallel group CRT, where certain district TB labs and their associated clinics were denied access to Xpert for an extended period of time [12, 24], (3) the phased rollout of Xpert provided logistical advantages, because it meant that a single site activation team, in charge of training and activation of the Xpert device, could sequentially initiate all study sites [24], (4) the need for only a single site activation team reduced projected study cost, (5) program managers and funders were interested in assessing accuracy of the Xpert diagnostic algorithm in a real-world environment rather than trying to assess accuracy in a tightly controlled research environment, with limited external validity [25], (6) in a real-world setting, the sequential rollout of an intervention allows lessons learned during earlier steps to be applied during later steps, and (7) a stepped-wedge design provides analysis options that allow for the control of trends over time [26, 27].

### Study design description

**Fig. 1:**
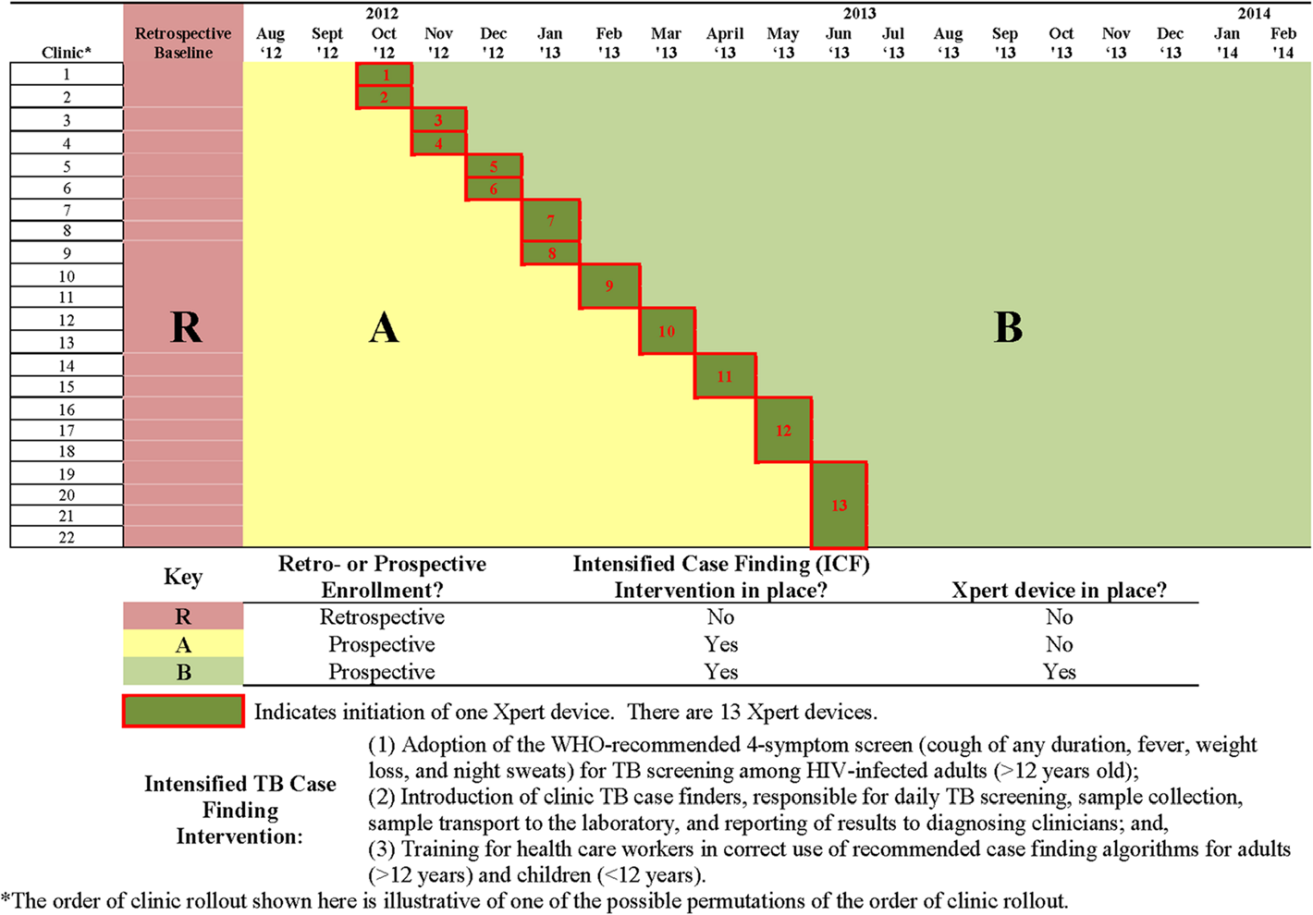
Study design for the Xpert package rollout evaluation using a stepped wedge design (XPRES)

**Fig. 2:**
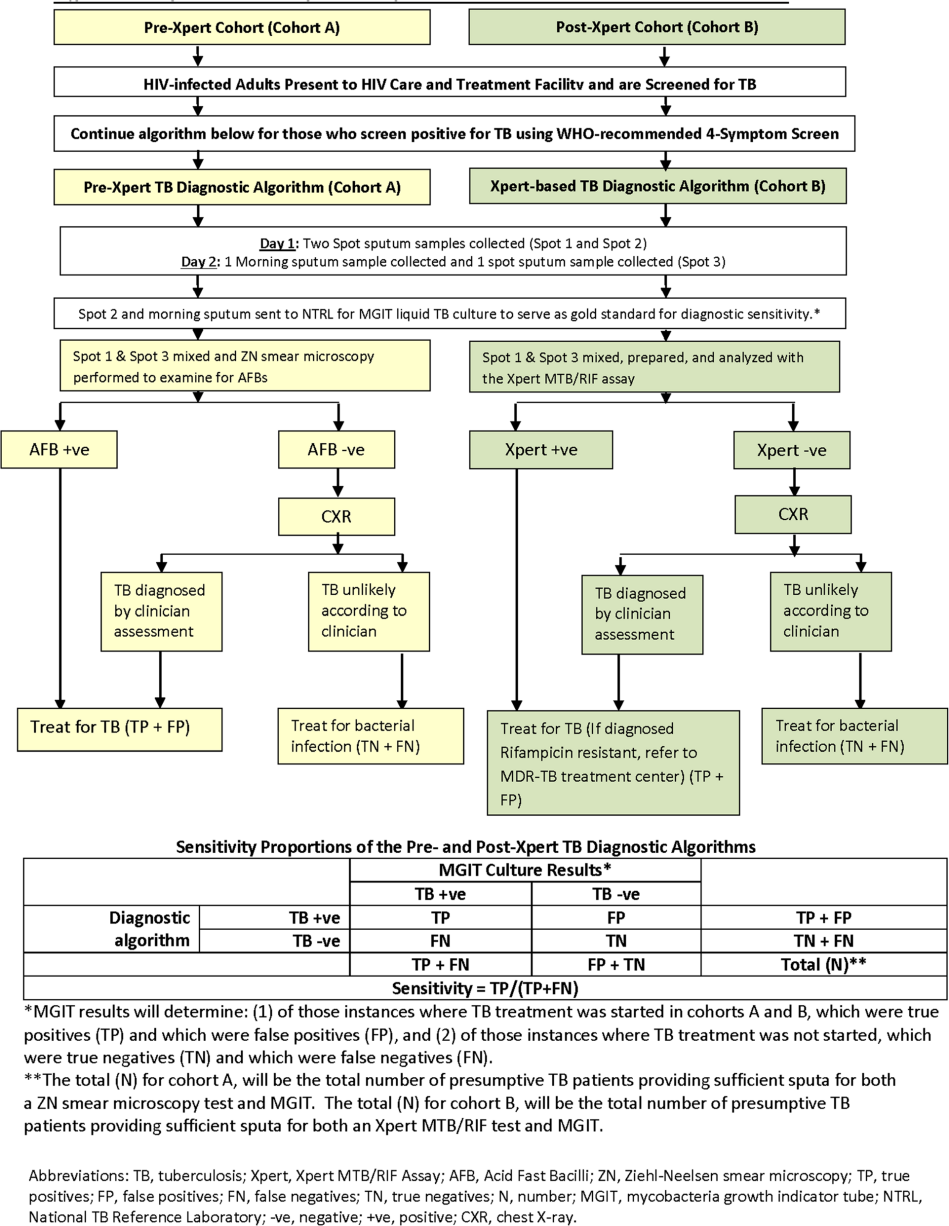
Comparison of pre-X pert and Xpert-based TB diagnostic algorithms in adults

**Fig. 3:**
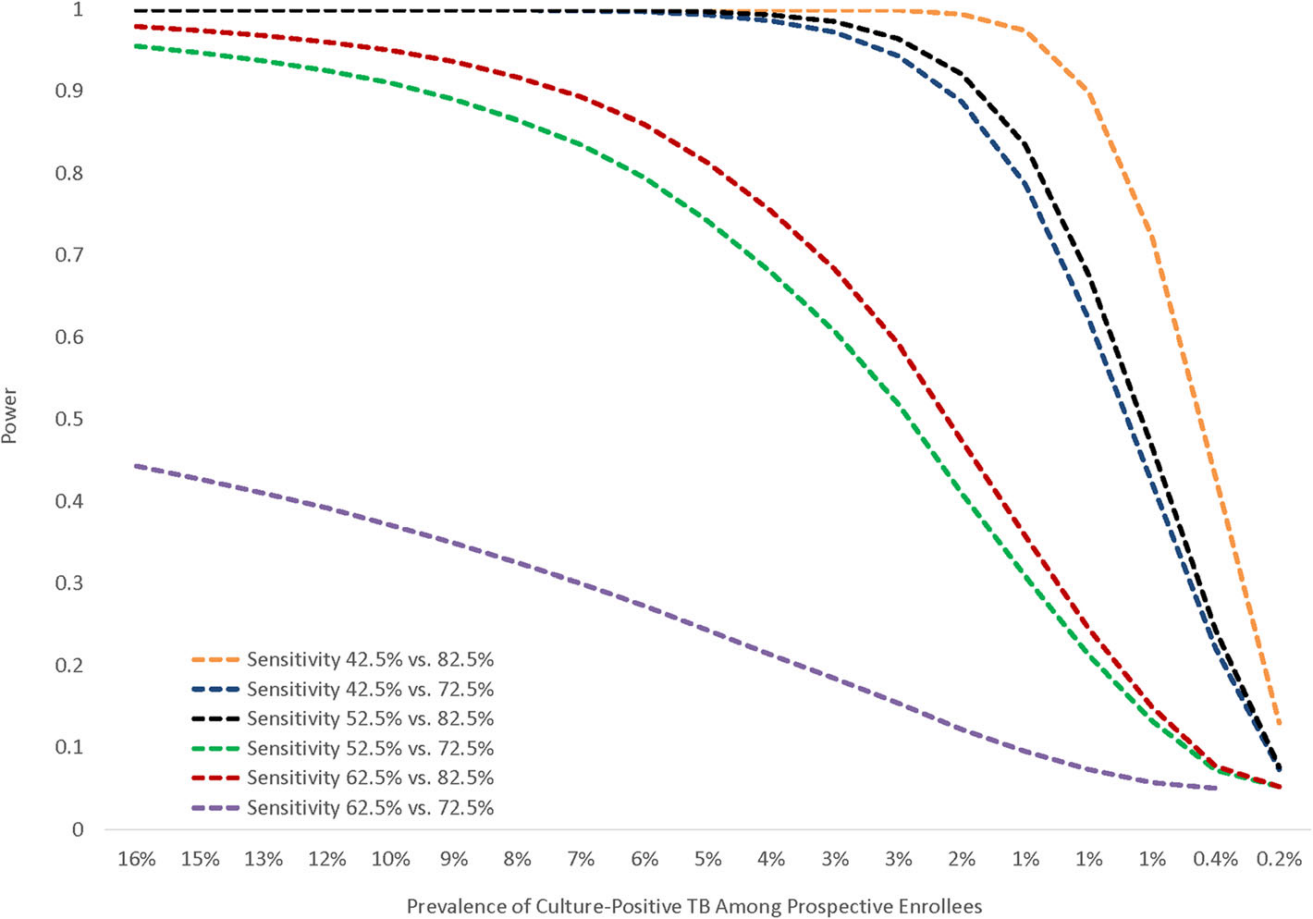
Figure showing power to detect a difference in TB diagnostic algorithm sensitivity pre- versus post-Xpert over a range of culture-positive TB prevalence rates at study enrollment according to actual prospective cohort sample size (*N* = 6,136)

Figure 1 summarizes the study design. This step-wedge design involved enrollment of three cohorts: (1) retrospective cohort (R), shaded in red in Fig. 1, (2) prospective cohort A (enrolled pre-Xpert device rollout), shaded in yellow in Fig. 1, and (3) prospective cohort B (enrolled post-Xpert device rollout), shaded in green in Fig. 1. For cohort R, all patients who initiated ART at one of the 22 HIV clinics for the first time in the 24 months before study start (i.e., before July 31, 2012), were eligible for enrollment. For cohort A, all patients who attended one of the 22 HIV clinics for the first time after study start (July 31, 2012), but before Xpert device rollout, were eligible for enrollment. For cohort B, all patients who attend one of the 22 HIV clinics for the first time after Xpert device rollout were eligible.

To answer the first primary study question, sensitivity of the pre-Xpert TB diagnostic algorithm in prospective cohort A will be compared with the post-Xpert algorithm sensitivity in prospective cohort B. Figure 2 describes the differences in TB diagnostic algorithms between cohorts A and B and how sensitivity proportions will be determined.

To meet the second key study objective (comparison of pre- versus post-Xpert package all-cause ART mortality), all-cause 6-month ART mortality rates will be compared between the retrospective cohort (cohort R) and the post-Xpert prospective cohort (cohort B). Since the cohorts being compared (cohorts R and B) do not overlap in a phased manner that would allow for controlling for secular trends according to analytic approaches recommended by Moulton et al [26] or Hussey & Hughes [27], this analysis approach is best characterized as a before and after comparison. However, secondary analyses, comparing 6-month ART mortality, and other ART outcomes, between cohorts A and B will make use of the stepped-wedge portion of the trial and analytic approaches recommended by Moulton et al [26] and Hussey & Hughes [27] to control for secular trends.

### Interventions

As described above, Fig. 2 illustrates the differences between the microscopy-based algorithm used in cohort A (pre-Xpert device rollout), and the Xpert-based algorithm used in cohort B (post - Xpert device rollout).

For the second key question, comparing all-cause 6-month ART mortality in cohort R with cohort B, Table 1 summarizes the differences between cohort R and B in terms of TB case finding and patient management activities. Notably, in addition to implementing ICF activities, study nurses were responsible for tracing prospectively enrolled patients (patients in cohorts A and B) who were ≥1 day late for a clinic appointment through up to five telephone calls and two home visits to return patients to HIV care. Indicators measuring compliance with all interventions, including implementation of the appropriate TB diagnostic algorithm and the tracing intervention, will help inform discussions of causal pathways during analysis of intervention impact on 6-month ART mortality.

**Table 1:** Comparison of TB case finding and patient management interventions for PLHIV in the retrospective and prospective cohorts

	Retrospective (R)	Prospective pre-Xpert (A)	Prospective post-Xpert (B)
TB screening algorithm for adults	1. Cough of any duration 2. Fever of any duration 3. Shortness of breath 4. Chest pain 5. Haemoptysis 6. Loss of appetite 7. Loss of weight 8. Malaise 9. Night sweats	1. Current Cough 2. Current Fever 3. Loss of weight 4. Night sweats	1. Current Cough 2. Current Fever 3. Loss of weight 4. Night sweats
Number of sputa collected from patients suspected of having TB	2 spot sputa	4 (2 spot sputa on day 1, 1 morning sputum on day 2, and one spot sputum on day 2)	4 (2 spot sputa on day 1, 1 morning sputum on day 2, and one spot sputum on day 2)
Adherence to TB screening algorithms	Estimated to be low	High	High
Specialized TB case finding nurses support TB case finding activities	No	Yes	Yes
Regular training for clinic personnel in ICF activities	No	Yes	Yes
Diagnostic algorithm in place	Microscopy + chest X-ray for smear-negative suspects	Microscopy + chest X-ray for smear-negative suspects	Xpert + chest X-ray for Xpert-negative suspects
Gold standard TB diagnostic test (MGIT) at national TB reference laboratory (NTRL)	Infrequent utilization of MGIT liquid TB culture at NTRL	MGIT liquid TB culture for all patients suspected of having TB. Prior to culture, fluorescent microscopy was conducted at NTRL.	MGIT liquid TB culture for all patients suspected of having TB. Prior to culture, fluorescent microscopy was conducted at NTRL.
TB drug resistance	Infrequent requests for TB drug resistance tests.	All positive MGIT TB cultures received: (1) LPA, (2) Phenotypic culture-based DST.	All positive MGIT TB cultures received: (1) LPA, (2) Phenotypic culture-based DST.
Patient tracing interventions in place	Irregular attempts to trace patients late for clinic appointments through telephone calls and home visits.	Tracing of patients late for clinic appointments through telephone calls and home visits.	Tracing of patients late for clinic appointments through telephone calls and home visits.

*Abbreviations*: *ICF* intensified TB Case Finding, *TB* tuberculosis, *MGIT* mycobacteria growth indicator tubes, *LPA* line probe assay, *DST* drug susceptibility testing

### Study population

#### Health facilities

The main reason for selecting the 22 facilities in Table 2 is that they are considered by study investigators and MOH to be representative of facilities in Botswana in terms of TB case finding capacity and ART service delivery. Study facilities consist of five district hospitals and 17 primary healthcare clinics (PHCs). Other advantages of choosing these facilities are: (1) on average they had anticipated high patient enrollment rates (at 33 patients/month/clinic), which helped meet the desired study power (see below), (2) one clinic (Gantsi, in Western Botswana) is estimated to have high prevalence of multi-drug resistant TB among HIV clinic enrollees and so could benefit from early rollout of the Xpert device per WHO recommendations [22], and (3) all 22 clinics had at least one year’s experience in providing ART services by the time of study start. Table 2 summarizes the study facilities chosen for XPRES.

**Table 2:** Selected study sites for the Xpert package rollout evaluation using a stepped-wedge design (XPRES)

District	Fixed consortiums	Clinic names	Pre-study estimates of no. new ART patients/month	Xpert location
Ngami (Maun)	fixed triplet	Letsholathebe II Memorial Hospital	36	1 × Lab Xpert
Ngami (Maun)		Boseja Clinic	28	
Ngami (Maun)		Maun Clinic	28	
Gaborone	fixed pair	Brodhurst Traditional Clinic	35	1 × Lab Xpert
Gaborone		Bontleng Clinic	45	
Francistown	fixed pair	Botswelelo Clinic	22	1 × Lab Xpert
Francistown		Area W Clinic	22	
Francistown	single facility	Nyangabgwe Referral Hospital	18	1 × Lab Xpert
Kweneng East-Molepolole	fixed quadruplet	Borakalalo Clinic	9	1 × POC Xpert
Kweneng East-Molepolole		Kgosing Clinic	8	
Kweneng East-Molepolole		Molepolole Central Clinic	8	
Kweneng East-Molepolole		Phuth-kobo Clinic	8	
Kweneng East-Mogoditsane	single facility	Nkoyaphiri Clinic	46	1 × POC Xpert
Palapye	fixed pair	Ext 3 Clinic	20	1 × POC Xpert
Palapye		Lotsane Clinic	20	
Bobirwa	single facility	Bobonong Primary Hospital	38	1 × Lab Xpert
Kanye	single facility	SDA Hospital	22	1 × Lab Xpert
Gantsi	single facility	Gantsi Clinic	unknown*	1 × Lab Xpert
Kgatleng	single facility	Deborah Memorial Hospital	26	1 × Lab Xpert
Lobatse	single facility	Athlon Clinic	18	1 × Lab Xpert
Serowe District	fixed pair	Kadimo Clinic	12	1 × POC Xpert
Serowe District		Serowe Clinic	12	
Total (22 clinics)			479	13
Average per clinic			23	

*Abbreviations*: *POC* point of care, *Xpert* Xpert MTB/RIF, *lab* laboratory, *ART* antiretroviral therapy

*Routine monitoring data on rate of enrollment of antiretroviral therapy patients was not available at the time of study initiation for this clinic

#### Study patients

For the retrospective cohort, all patients starting ART in the 24 months before study start, except for prisoners, were eligible for chart abstraction to estimate all-cause ART mortality rates. For the prospective cohorts (A and B), all patients, who met consent requirements, registering for HIV care for the first time at the facility in the 19 months after study start, were eligible for enrollment, except for prisoners. Prisoners were excluded because it would be difficult to obtain comprehensive retrospective cohort data for cohort R, due to frequent unscheduled prisoner movement during incarceration, and difficult to retain prisoners in cohorts A and B for the study’s duration. Children (<12 years of age) were eligible for prospective enrollment, if their assent and guardian’s consent were provided, because secondary study questions aim to estimate Xpert algorithm sensitivity for children and its impact on pediatric ART outcomes.

### Randomization procedures

Because some of the clinics use the same TB diagnostic facility, Xpert activation was simultaneous for these clinic consortiums (Table 2). For scheduling purposes and because clinic staff rotations occurred at the end of calendar months, each step in the stepped-wedge design needed to be equivalent to one calendar month. In addition, because the MOH and partners wanted the 13 Xpert devices to be operational and serving patients in nine months rather than 13 months, there was a need to initiate two Xpert devices during a single step for four of the nine steps. Taking into consideration constraints in Xpert device assignments to clinics (see Table 2 and Fig. 1), there were 9! (362,880) possible permutations of the order of Xpert rollout. The study statistician randomly selected one of these permutations [28].

### Sample size and power—first key objective

Funding availability limited the prospective enrollment duration to 19 months at the 22 study facilities. MOH monitoring data reported an average of 23 new ART-eligible patients enrolling in each facility per month and investigators estimated there were 10 new ART-ineligible patients enrolled per month at each facility (i.e., potentially 33 study-eligible patients/month/clinic). To be conservative with sample size estimates, we assumed that only 70 % of study-eligible patients would agree to prospective enrollment in the study (i.e., 23 study patients/month/clinic), giving anticipated cohort A and B sample sizes of 3,266 and 6,348, respectively (*N* = 9,614). Since the vast majority of patients (>99 %) at these study clinics were adults (≥12 years old), for the purpose of sample size calculations, we assumed all 9,614 prospective enrollees would be adults.

Based on a published meta-analysis, we estimated that about 49 % of new adult HIV clinic enrollees would screen positive for TB [29], and that about 33 % of those screening positive would have culture-confirmed TB, giving an overall active TB prevalence among adult study enrollees of about 16.2 % [30–35]. Our literature review suggested true active TB prevalence among adult PLHIV entering HIV care ranged from 7.1 % in Ethiopia [34] to 31.5 % in South Africa [33]; since Botswana has a higher TB case notification rate (about 470/100,000 population) than Ethiopia (about 224/100,000 population) but a lower TB case notification rate than South Africa (about 860/100,000 population), our estimate of adult active TB prevalence of 16.2 % at HIV care entry in Botswana was considered reasonable [36–38]. Further literature review suggested that the pre-Xpert, microscopy-based TB diagnostic algorithm sensitivity might be as high as 62.5 % [34], and that Xpert algorithm sensitivity among symptomatic PLHIV could be about 82.5 % based on data from a recent multi-country Xpert accuracy study [12].

To estimate power, data were simulated according to the stepped-wedge design, using the beta-binomial model to induce the intra-cluster correlation coefficient. One thousand datasets were simulated and a mixed model appropriate for the stepped-wedge design fit to the data, as described by Hussey and Hughes [27], that included fixed effects for time and intervention condition (0 for time points before Xpert implementation and 1 afterward), and a random effect for the clinic, to take into account between-clinic variability. For protocol-specified sample sizes (*N* = 9,614), and assuming culture-positive TB prevalence of 16.2 % at study entry, and pre-versus post-Xpert sensitivity comparisons of 62.5 % vs. 82.5 %, we had 99.6 % power. In multiple simulations, pre-Xpert sensitivity was varied from 55 % to 62.5 % and post-Xpert sensitivity from 70 % to 82.5 % and the study had >80 % power to detect the intervention effect across all simulated scenarios.

After study initiation, monitoring data, prepared by study nurse supervisors during supervision visits, revealed that actual monthly HIV clinic enrollment numbers were lower than expected (about 21 patients/clinic/month instead of 33 patients/clinic/month). In addition, study nurses were only able to enroll about 72 % of study-eligible patients at the clinic, mostly because patients were not willing to wait while the study nurse completed enrollment of other patients, a process which took about 1 h/enrollee. Therefore, prospective enrollment occurred at about 15/month instead of the protocol-specified 23/month. In addition, the proportion of adult patients screening positive for TB at prospective cohort enrollment was lower than expected (24 % instead of 49 %), and the proportion of those screening positive, who were diagnosed with culture-positive TB, was lower than expected (17 % instead of 33 %), giving a much lower culture-positive TB prevalence at enrollment than was originally expected (about 4 % instead of 16 %).

Lower numbers of culture-positive TB cases/clinic/month (1/clinic/month instead of 4/clinic/month) resulted in inability to fit the stepped-wedge model to all simulated datasets. Therefore, the power estimation approach was simplified and Fisher’s Exact Test for comparing two proportions (pre-and post-Xpert) in SAS version 9.2. software (SAS Institute Inc., Cary, NC) was used to estimate study power. Power estimates were then adjusted for the expected design effect to account for intra-cluster correlation. For design effect calculations, an intra-class correlation of 0.05 was assumed [28]. Input estimates for TB prevalence at HIV care enrollment and TB diagnostic sensitivity were varied to understand impact on study power (Fig. 3). As illustrated in Fig. 3, sample size and TB prevalence shortfalls meant we only had about 75.4 % power to detect the protocol-specified difference in sensitivity (62.5 % vs. 82.5 %) if active TB prevalence at enrollment was 4 %. However, we would have >80 % power to detect pre-versus post-Xpert TB diagnostic sensitivities at a culture-positive TB prevalence of 4 % if pre-Xpert sensitivity was ≤52.5 % and post-Xpert sensitivity ≥82.5 % (Fig. 3). Because available National TB Reference Laboratory (NTRL) monitoring data suggested pre-Xpert TB diagnostic sensitivity was ≤52.5 % and Xpert sensitivity ≥82.5 %, the study was considered still well powered to answer the first key study question at quarterly reviews conducted by the study sponsor.

### Sample size and power—second key objective

To estimate power for the comparison of all-cause 6-month mortality in the retrospective cohort (cohort R) versus the post-Xpert prospective cohort (cohort B), the approach of Moulton et al, suitable for stepped-wedge trial designs, was chosen because these power estimates were more conservative than those derived from a pre-post sample size calculation [26]. Per this approach, published formulae for the comparison of two rates in an unmatched parallel group CRT [39] were adapted to the stepped-wedge design as follows:

$$ {Z}_{\beta } = \sqrt{\frac{\left(c-1\right){\left({r}_c-{r}_t\right)}^2\ }{\left[{r}_0/{y}_c + {r}_1/{y}_t+{k}^2\left({r}_0^2 + {r}_1^2\right)\right]}} - {Z}_{\alpha /2} $$

where *Z*_*β*_ is the standard normal deviate corresponding to the upper tail probability of *β* and *β* is the probability of a Type II error; *c* is the number of clusters (study facilities) per arm, where, since this is a stepped-wedge trial involving 22 clinics, 22/2 was used [26]; *r*_*c*_ is the estimated true 6-month ART mortality rate in the pre-intervention control phase (cohort R); *r*_*t*_ is the estimated true mortality rate in the post-intervention phase (cohort B); *y*_*c*_ is the average number of person-years (PYs) per clinic in the control phase, estimated as the average retrospective cohort size per clinic (552) divided by two since each patient commits 6 months of follow-up time to the analysis; *y*_*t*_ is average number of PYs per clinic in the intervention phase, conservatively estimated as the harmonic mean of PYs contributed by each study site in cohort B, again assuming 6 months of follow-up time per participant [26]; *k* is the estimated between-cluster coefficient of variation of the true rates in both the control and intervention phases, estimated as 0.2 [26]; *Z*_*a/2*_ is the standard normal deviate corresponding to the upper tail probability of *a*/2 where *a* is the probability of a Type I error.

Since a log-rank test statistic for intervention effect calculated for a simulated stepped-wedge trial (*Z*_*SW*_) will generally always be lower than the corresponding statistic (*Z*_*E*_) for a parallel group trial, because allocation ratios of patients to intervention or control status for parallel group trials remain equal while for stepped-wedge trials they are usually unequal, except at the mid-point of the stepped-wedge design, the z-score in the stepped-wedge trial formula (*Z*_*β*_ above) was divided by a published estimate of *Z*_*E*_/*Z*_*SW*_ (i.e., 1.2) prior to extrapolating the z-score to a power estimate [26]. Similarly, for Type 1 error of 5 %, instead of assuming a *Z*_*α*/2_ of 1.96, an inflated estimate of 2.352 was used, per published precedent [26].

**Fig. 4:**
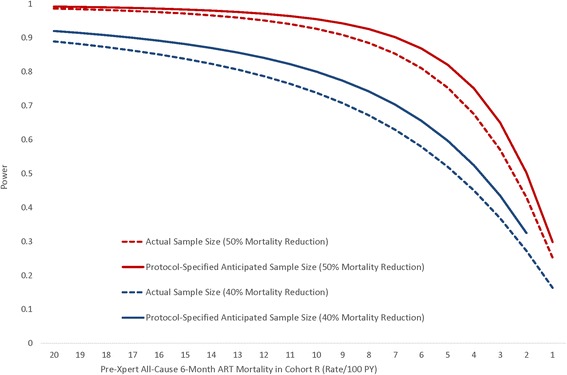
Power to detect a 40 % and 50 % difference in all-cause 6-month ART mortality between pre-Xpert retrospective and post-Xpert prospective cohort enrollees over a range of pre-Xpert mortality rates

Prior to study start, available data from Botswana suggested that the documented all-cause early mortality rates in the first 6 months of ART among adults were about 15 deaths per 100 PYs [40], which was similar to estimates from a meta-analyses of 18 programs in RLS with active tracing programs (14.7/100 PY) [41]. Since Botswana data, and available meta-analyses suggested about 40 % of deaths among PLHIV were due to undiagnosed TB or TB diagnosed late, and given that interrupting ART during the first 6 months of therapy by missing clinic appointments increases mortality risk [42, 43], it was considered reasonable that the Xpert package plus the tracing intervention might reduce mortality by about 40 % [2, 44]. According to protocol-specified sample sizes, the study had >80 % power to detect a difference in 6-month all-cause ART mortality between cohorts R and B of 40 % if cohort R mortality was ≥10/100 PYs (Fig. 4). According to anticipated actual sample sizes, the study has >80 % power to detect a difference in 6-month all-cause ART mortality between cohorts R and B of 40 % if cohort R mortality is ≥13/100 PYs (Fig. 4).

### Study procedures related to first key objective

For prospective cohort enrollment, all new HIV clinic enrollees were informed about the study, and if interested, offered the opportunity to enroll following the Institutional Review Board (IRB)-approved consent process. An enrollment questionnaire collected important baseline demographic and clinical information, and a patient locator form was used to document telephone numbers and home addresses for patient retention activities. Adult patients screening positive for TB were asked to provide four sputa, two collected simultaneously the day of enrollment (referred to as “spot” sputa), and two the following day. Of the two sputa provided the second day, one was a morning sputum prepared by the patient soon after waking up, and the other a spot sputum provided upon arrival at the clinic. Additional file 1 shows a poster used by study nurses to inform the patient how to produce a good sputum. The poster also illustrates important infection control precautions (e.g., preparing spot sputa in a well-ventilated, but still private, “cough spot” outside the clinic). If a patient screening positive for TB was unable to spontaneously produce sputa, MOH-recommended sputum-induction procedures were encouraged if there were no contra-indications [23]. For children <12 years old, who were unable to produce sputa spontaneously and were too young for induction (i.e., <5 years old), naso-gastric tube aspirates were recommended per MOH guidelines [23]; however, very few children <5 were expected to enroll in the study.

Spot sputa numbers one and three were sent to the on-site or peripheral district TB lab for: (1) smear microscopy, and (2) Xpert, if the Xpert device had been activated by that time. Spot sputum 2 and the morning sputum were sent to the NTRL for: (1) fluorochrome acid fast bacilli (AFB) smear microscopy on concentrated specimens, (2) liquid culture in mycobacteria growth indicator tubes, (3) confirmation of any mycobacterial growth as *Mycobacterium tuberculosis* (MTB) or non-tuberculous mycobacteria through Ziehl-Neelsen staining, blood agar plate, and immunochromatographic assays, (4) line probe assay testing for isoniazid and rifampicin resistance on MTB-positive cultures, and (5) phenotypic culture-based drug susceptibility testing on MTB-positive cultures. All test results were returned to study nurses with recommended maximum turnaround times from the time of sample collection to result return to the nurse being four days for smear microscopy at the peripheral lab, 10 days for flourochrome smear microscopy at the NTRL, two days for Xpert testing regardless of Xpert location, and 49 days for liquid culture results from the NTRL. Nurses were encouraged to inform patients of positive TB diagnoses the same day via phone, although, if the patient was unreachable by phone, the patient was informed at the next scheduled clinic appointment.

For the first 17 months of study conduct, all prospectively enrolled patients consented to 12 months of follow-up but this was shortened to 6 months of follow-up in December 2013 (17 months into study enrollment), in an attempt to reduce burden on study nurses.

### Study procedures related to second key objective

Enrollment of the retrospective cohort (cohort R) was through chart abstraction of eligible patients who started ART in the 24 months before study initiation at one of the 22 study clinics. Chart abstraction procedures were similar to procedures described in previous studies [45]. Data on important demographic and clinical characteristics were abstracted to maximize opportunities to explore and control for confounding, when estimating impact of the Xpert package on all-cause mortality. Since loss to follow-up (LTFU) from ART can account for as much as 75 % of all attrition (death plus LTFU) in ART programs, and because incidence of death following LTFU ranges from 20 % to 60 % and failure to adjust mortality estimates for death among LTFU patients could bias estimates of intervention effect [40, 46], tracing patients LTFU was considered essential to answer the second key question. Tracing for mortality ascertainment purposes, was conducted through two methods in all cohorts. Firstly, up to five telephone calls and two home visits were used to determine outcomes of patients LTFU; in the retrospective cohort this occurred following documentation that the patient was >90 days late for a scheduled appointment, whereas in the prospective cohort this tracing started the day following a missed appointment. Secondly, all patients that remained LTFU following telephonic and home visit tracing activities were searched for in Botswana’s national mortality database.

Since about 2,429 (28 %) of 8,565 patients eligible for the prospective cohort did not enroll due to logistical constraints (see above), a protocol amendment was approved in April 2014, that allowed retrospective chart abstraction of the missed prospective patients. By abstracting key baseline data (e.g., baseline CD4 count) and outcome data (e.g., vital status) on the 2,429 missed patients, investigators will be able to: (1) estimate if the prospective cohort is truly representative of new HIV clinic enrollees at the 22 study sites during study conduct, and (2) use appropriate methods to explore effect of non-response [47].

### Analytic methods

For the first key study question, we will employ a mixed-model approach similar to that presented by Hussey and Hughes (2007) [27]. A generalized linear mixed model will be fit to the data. The dependent variable is dichotomous, indicating whether the diagnostic algorithm detected TB or not (only those with true TB detected by liquid culture will be included in the sensitivity analysis). A fixed effect for time will be included in the analysis to adjust for any time trends that might bias the pre-post comparison. A fixed effect for intervention condition (0 before Xpert implementation, 1 afterward) will also be included and is the test of the intervention effect. A random effect for clinic will be included to adjust for between-clinic variation. The intervention effect will be judged significant at p < 0.05 with a two-tailed test.

For the second key question, crude and multivariable Cox proportional hazards regression models, accounting for study design, will be fit to the data with a fixed effect specified for intervention status, and random effect for clinic [28]. Since there are three levels to the intervention, with cohort R receiving standard of care, cohort A receiving the ICF intervention, and cohort B receiving the ICF intervention plus the Xpert diagnostic algorithm, intervention effect will be coded as a three-level variable to represent the three study phases. Although the protocol-specified primary question aims to compare 6-month mortality in cohort R versus cohort B, investigators will examine for any dose-response effect across cohorts R, A, and B, which could add data to inform interpretation of causal pathways [48]. Importantly, the analysis will need to control for trends over time. Several reports from RLS, including Botswana [49], have reported improvements in baseline health status at ART initiation (e.g., higher median CD4 counts) and lower incidence of 6-month ART mortality over successive annual cohorts of ART enrollees. As described earlier, because cohorts R and B do not overlap during the stepped-wedge portion of this trial, a comparison of 6-month mortality rates in cohorts R and B cannot make use of the stepped-wedge design to control for secular trends [26]. However, since most variation in 6-month ART mortality over successive annual ART cohorts is accounted for by changes in health status of ART enrollees (e.g., changes in baseline CD4 count), incorporation of these known risk factors for 6-month ART mortality into the multivariable model may fully account for secular mortality trends [7, 50].

In a secondary analysis, that excludes cohort R, we will compare 6-month ART mortality rates between cohorts A and B using analytic methods described by Moulton et al, fitting Cox proportional hazards models to the data with the underlying time frame being time since July 2012 (initiation date for the stepped-wedge component of the trial), fixed effect for intervention arm (Xpert device activation), and a random effect for clinic [26, 51]. We will also explore an alternate analytic approach, recommended by Hussey & Hughes, which utilizes a Poisson model, including fixed effect for intervention and time interval, and a random effect for cluster [27].

### Ethical considerations

This research study was reviewed and approved by the CDC IRB C, the Health Research and Development Division of the Human Resource Development Council (HRDC) in Botswana, and the University of Pennsylvania IRB No.4. XPRES is registered at ClinicalTrials.gov (trial registration no. NCT02538952).

### Trial status

Prospective cohort enrollment started in July, 2012 and was completed by the end of March 2014. Retrospective cohort chart abstraction was complete by December 2015. Data entry is estimated to be complete by the end of September 2016. Data analysis for the primary study questions has not yet begun. Trial data will be reported according to published guidelines for cluster-randomised trials (Additional file 2).

## Discussion

The over-arching purpose of this project is to improve TB diagnostic and care services at 22 HIV care and treatment clinics through phased rollout of (1) strengthened ICF systems, and (2) 13 Xpert devices, while simultaneously answering important implementation science questions, concerning Xpert operationalization and impact.

The stepped-wedge study design was chosen for a number of reasons related to ethical, operational, and analytic needs, as described in the method’s section. During the course of study implementation, the operational advantages of the phased implementation approach have been particularly notable. In our RLS of Botswana, the phased implementation approach has allowed the limited human and financial resources to be focused on smaller, more manageable pieces of the whole project, one step at a time, rather than be spread thinly across study sites, as would be required in a parallel group CRT [19]. Analytically, the stepped-wedge design allows multiple opportunities for controlling trends over time [26]. Potential disadvantages, when compared with a parallel group CRT, include: (1) moderately lower ability to assign causality to the intervention, and (2) higher sample size requirements in most circumstances, because of unequal allocation ratios for most of the duration of stepped-wedge trials [19]. The ethical, operational, and analytic advantages may help explain the increasing popularity of the stepped-wedge evaluation design, especially in RLS [24].

During trial conduct, several operational challenges were experienced, mainly related to lower than expected clinic enrolment rates, human resource constraints that reduced ability to enroll all study-eligible patients in the prospective cohort, and lower than expected prevalence of culture-positive TB at clinic enrollment. The declining HIV clinic enrolment rates probably reflect success of the HIV treatment program in reaching HIV-infected persons in prior years (i.e., during 2002–2011) [49], declining HIV incidence rates [52], and expanding numbers of alternate HIV clinics at which patients can receive care [49]. The study team probably over-estimated the willingness of patients to wait at the clinic for their turn to enroll in the study. However, in response to the observation that 28 % of potentially study-eligible patients were not being enrolled in the prospective cohort, the study team wrote a protocol amendment that allowed retrospective chart abstraction for the missed prospective patients, which will allow investigators to quantify any potential selection bias incurred by non-response. The lower than expected prevalence of culture-positive TB at HIV clinic enrollment needs further investigation once all study data are available for analysis. Fueled by the HIV epidemic, TB case notification rates in Botswana increased from about 202/100,000 population in 1990 to about 600/100,000 in 1998, plateaued at this level during 1998 through 2007, and have since declined to about 470/100,000 in recent years [36]. Increased ART coverage among HIV-infected persons might again explain declining national TB incidence and the lower-than-expected TB prevalence among HIV clinic enrollees in this study [53]. In retrospect, the protocol-specified large sample sizes and resulting high pre-study power to answer the first two primary study questions, were important precautions in place to ensure any sample size shortfalls did not result in trial futility.

Although, several Xpert impact studies have been published after this trial started, the two key study questions have not yet been answered. Firstly, data validating the Botswana Xpert diagnostic algorithm have not yet been reported, and this is an important program evaluation activity [54]. Secondly, among six trials that have compared all-cause mortality outcomes of study enrollees between microscopy and Xpert arms [17, 21, 54–57], none have observed Xpert impact on either morbidity or mortality outcomes, and only one was conducted exclusively among ART enrollees (Mupfumi et al) [21]. Certain study limitations of the trial by Mupfumi et al, including small sample size (*N* = 424) and powering the study to detect differences in a composite outcome (death or TB) between study arms, mean that XPRES, with its larger sample size (*N* = 16,267) and powered to detect Xpert impact on 6-month mortality rates specifically, is still positioned to provide a valuable scientific contribution. In addition, the intervention in XPRES is different from interventions employed in previous Xpert impact trials [17, 21, 54–59]—it represents a package of strengthened ICF interventions, activation of the Xpert device, and improved tracing for patients late for ART clinic appointments. In real-world settings, ICF interventions are often implemented at a sub-optimal level of quality and consistency due to health system weakness [9], and strengthening health systems to improve ICF compliance is arguably as important as the rollout of a new TB diagnostic device [17]. In addition, preventing treatment interruptions or LTFU during early ART through the tracing intervention, could contribute to reductions in all-cause, 6-month ART mortality rates [42, 43].

## Additional files

Additional file 1: Study Poster of Steps to Getting a Good Sputum Sample. (PDF 804 kb)
Additional file 2: CONSORT 2010 checklist of information to include when reporting a cluster randomised trial–XPRES Trial Checklist. (PDF 392 kb)

## Abbreviations

AFB: Acid fast bacilli
ART: Antiretroviral therapy
CDC: United States centers for disease control and prevention
CRT: Cluster-randomized trial
HCW: Healthcare workers
HRDC: Human resource development council
IRB: Institutional review board
LTFU: Loss to follow-up
MOH: Ministry of health
MTB: *Mycobacterium tuberculosis*
NTRL: National TB reference laboratory
PHC: Primary healthcare clinic
PLHIV: Persons living with HIV
PY: Person-years
RLS: Resource-limited settings
SAS: Statistical analysis software
TB: Tuberculosis; intensified tuberculosis case finding (ICF)
WHO: World Health Organization
Xpert: Xpert MTB/RIF assay
XPRES: Xpert package rollout evaluation using a stepped-wedge design
