# Supplementary material for: Implementation of a pragmatic, stepped-wedge cluster randomized trial to evaluate impact of Botswana’s Xpert MTB/RIF diagnostic algorithm on TB diagnostic sensitivity and early antiretroviral therapy mortality
Source: BMC Infect Dis. 2016 Oct 26;16:606. doi: 10.1186/s12879-016-1905-4 (PMC5080709; doi:10.1186/s12879-016-1905-4)
Supplement: Additional file 1: — Study Poster of Steps to Getting a Good Sputum Sample. (PDF 804 kb) [file 12879_2016_1905_MOESM1_ESM.pdf]

Steps to Getting a Good Sputum Sample:

1. Go to the sputum collection area you are directed to by the study nurse. This area should be outside or in a special area with negative air flow (meaning the air flows out of the room you are in).

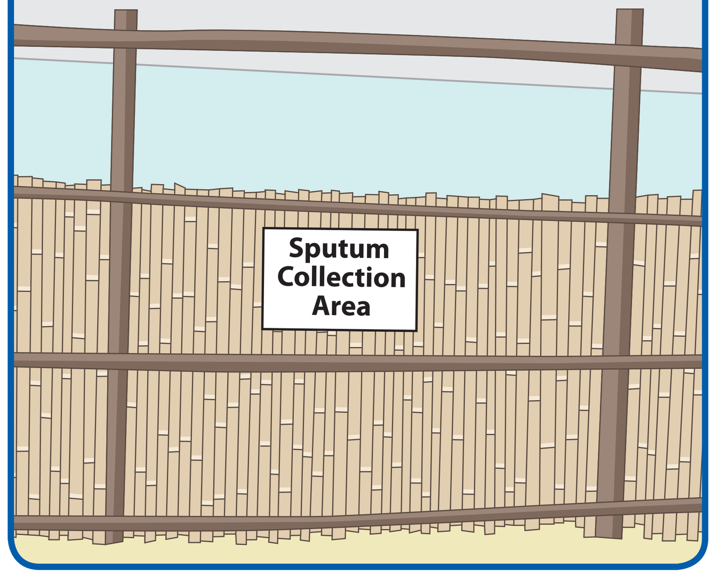

2. Relax. Take a few deep breaths while pressing your hand lightly on your stomach. When it's time to cough, you should be inhaling so deeply that you feel it in your stomach area.

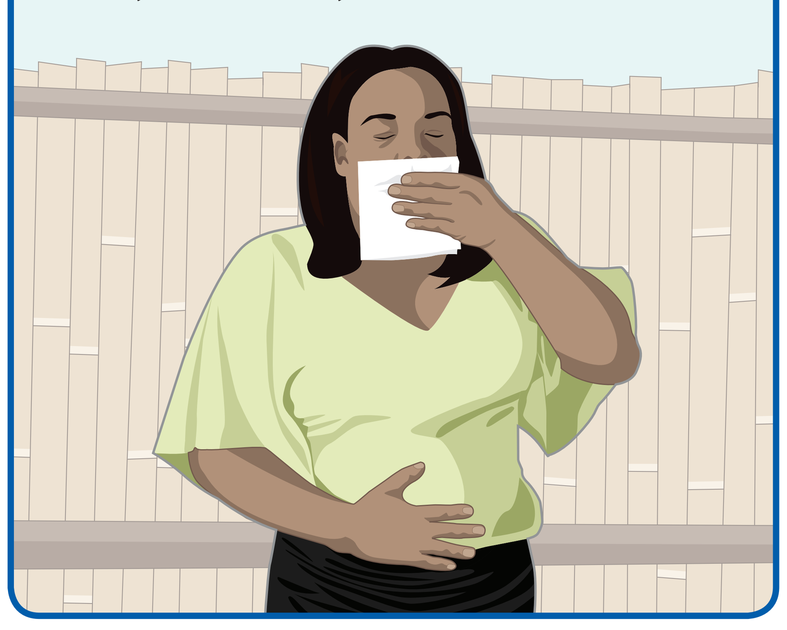

3. Rinse and spit with water. This is important to make sure there won't be mouth bacteria in the sputum collected.

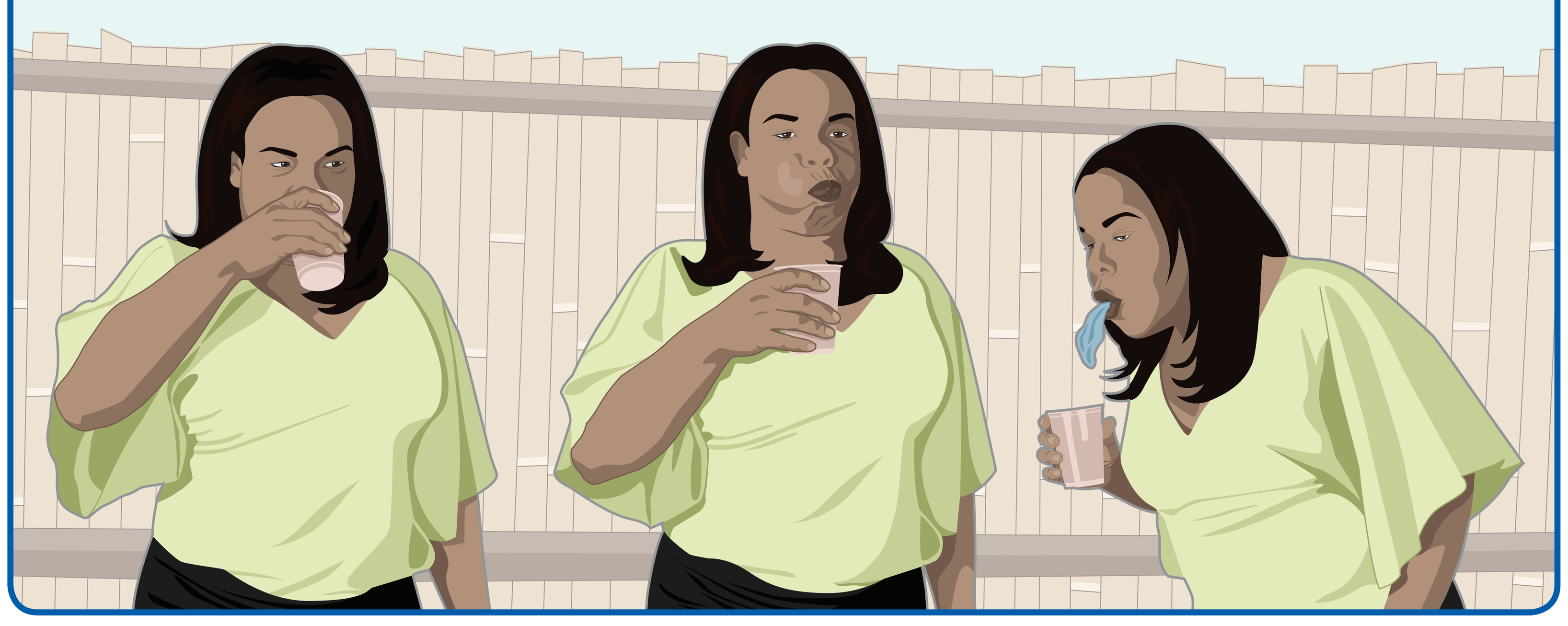

4. Get ready– Put one hand over your mouth with a tissue and then put the other hand back on your stomach.

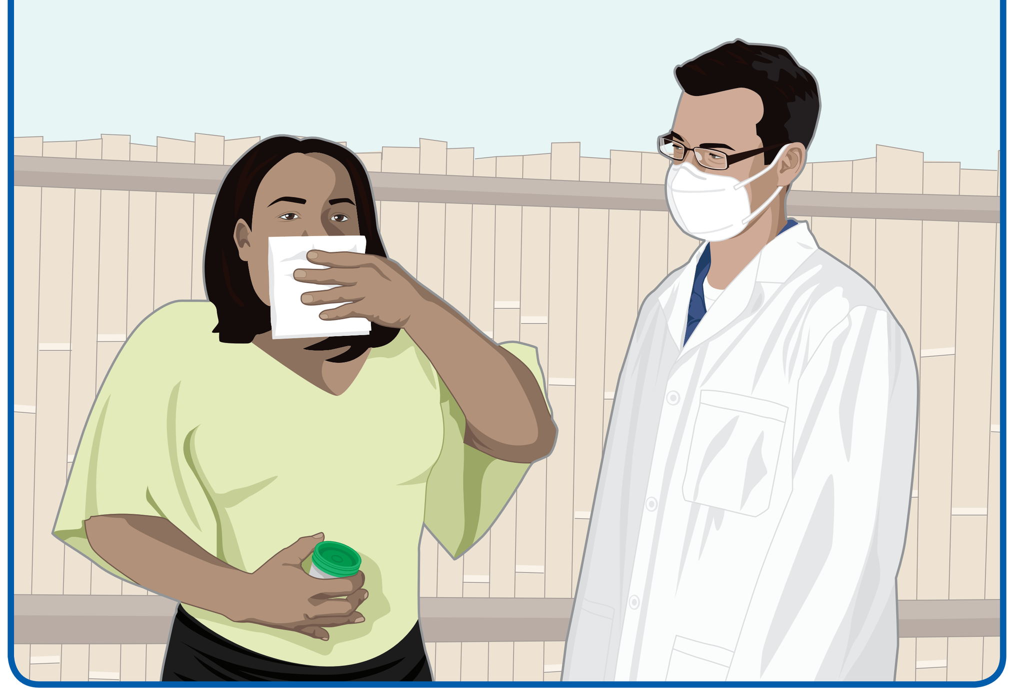

5. Cough deeply, so that you can really feel it in your stomach. Do not take shallow coughs from the throat or chest.

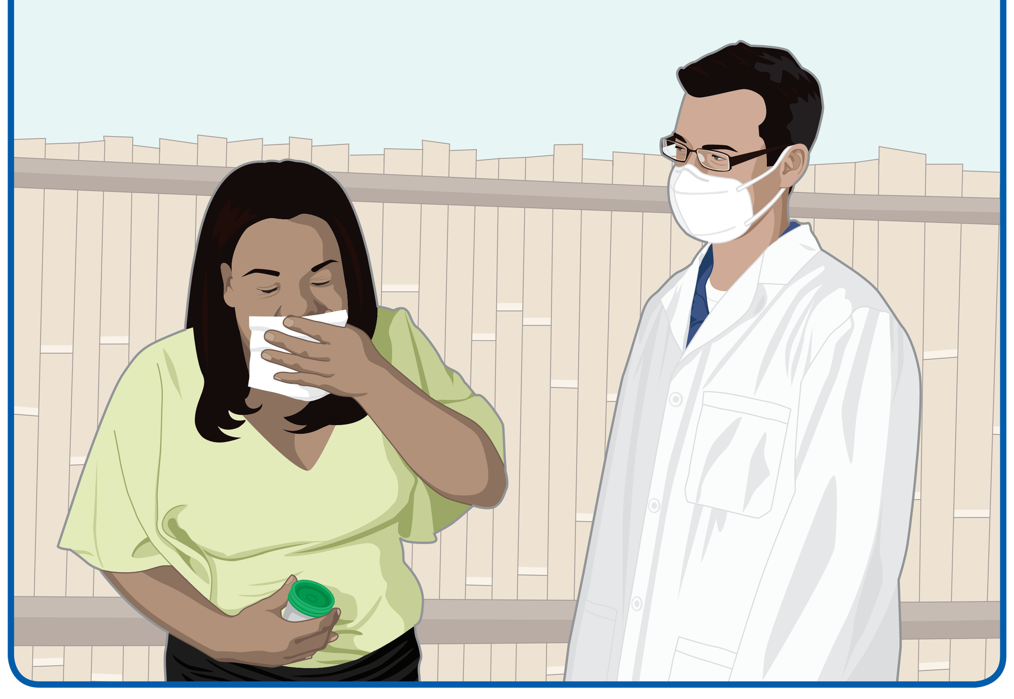

6. Once the sputum (phlegm) is in your mouth, **release** it into the container provided by the study nurse.

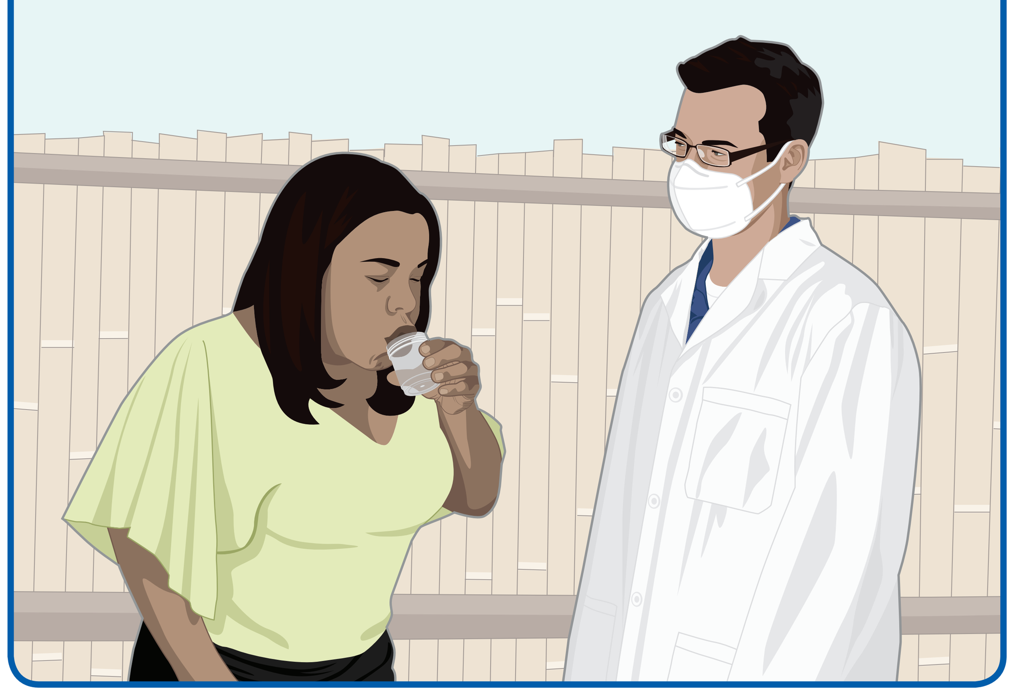

7. Check– Give the sputum (phlegm) container to the study nurse to check for quality. The study nurse should hold the container up to the light to make sure you have provided a quality sample.

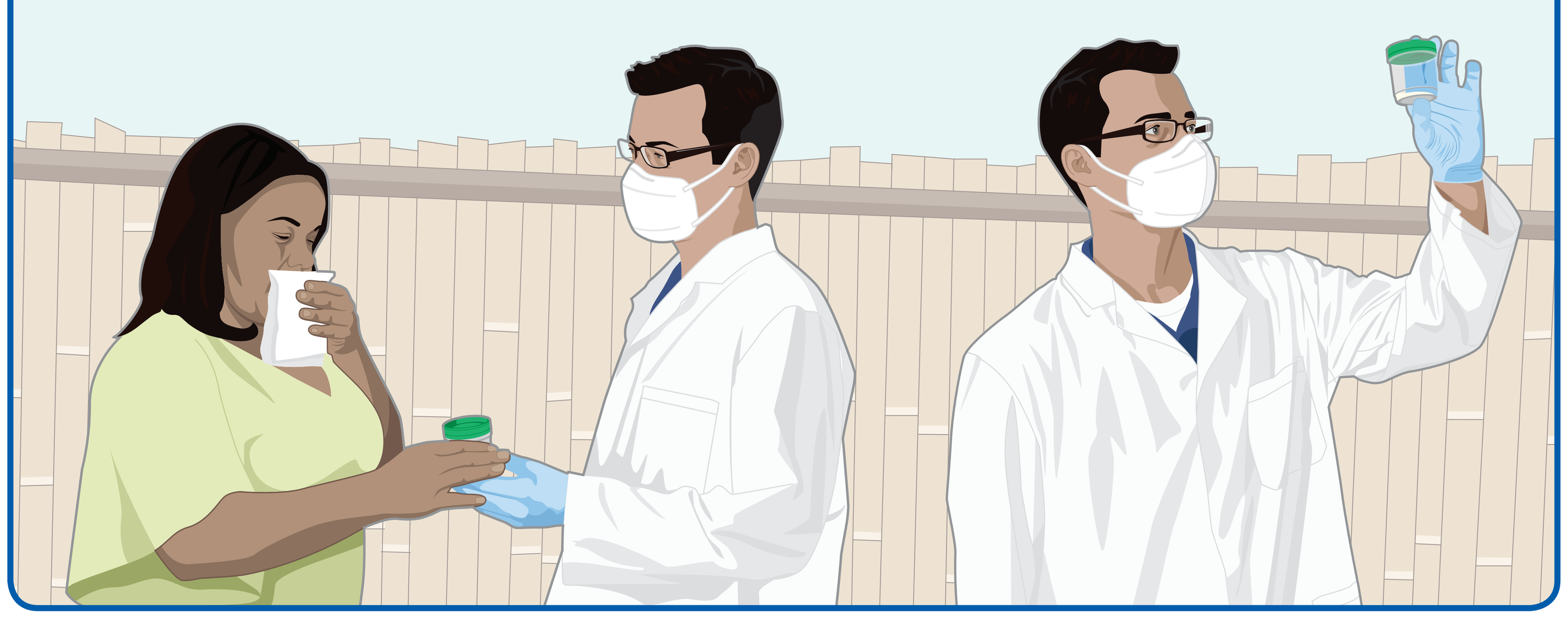

8. Repeat– On day 1, you need to produce **two** high quality sputa for the study nurse. If either of the two sputa are of low quality (too small or clear), according to the study nurse:

1. You will be asked to try to produce a better sputum. The study nurse will accompany you to the sputum collection area and will coach you through steps 1-8.

2. If the sample is still **too small** or is clear, the study nurse may assist you by patting you on the back (see below). Stretching will also help (see below).

3. If the samples you provide are good, you are done for the day, but you will need to come back to repeat these steps the following day (day 2) as directed by the study nurse.

If you are having trouble:

1. Get a pat! A study nurse may pat you solidly up and down your back to help you release the sputum.

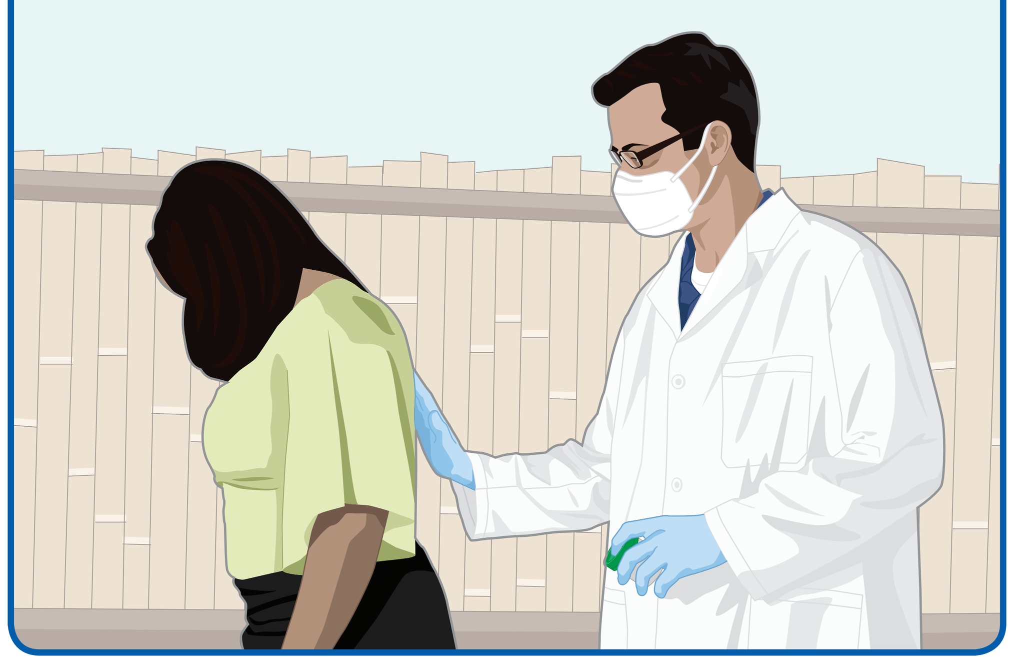

2. Stretch and try again– sometimes it helps to relax a bit. When you are ready, rinse and spit and try again!

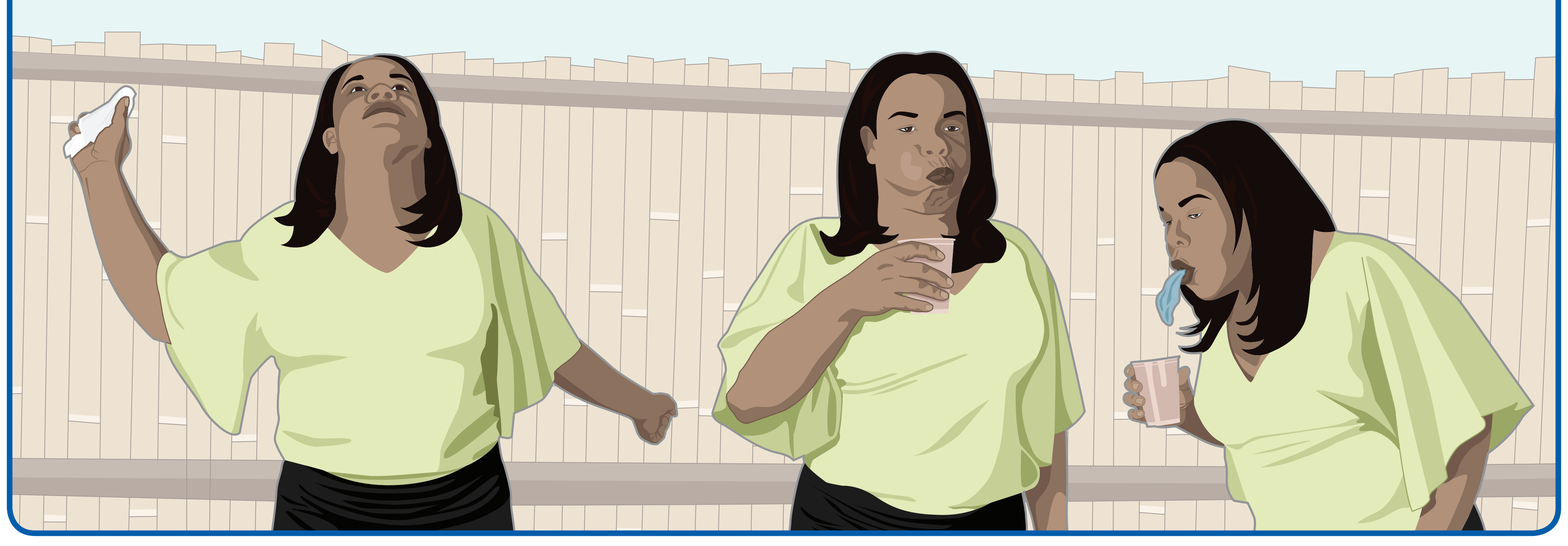

U.S. Department of Health and Human Services
Centers for Disease Control and Prevention

CS241782
